# Quantifying the inhibitory effect of Bcl‐xl on the action of Mff using live‐cell fluorescence imaging

**DOI:** 10.1002/2211-5463.12739

**Published:** 2019-11-15

**Authors:** Yunyun Ma, Mengyan Du, Fangfang Yang, Zihao Mai, Chenshuang Zhang, Wenfeng Qu, Bin Wang, Xiaoping Wang, Tongsheng Chen

**Affiliations:** ^1^ MOE Key Laboratory of Laser Life Science and Institute of Laser Life Science College of Biophotonics South China Normal University Guangzhou China; ^2^ Department of Pain Management The First Affiliated Hospital Jinan University Guangzhou China

**Keywords:** apoptosis, Bcl‐xl, FRET two‐hybrid assay, living cells, Mff, stoichiometry

## Abstract

Mitochondrial fission regulates mitochondrial function and morphology, and has been linked to apoptosis. The mitochondrial fission factor (Mff), a tail‐anchored membrane protein, induces excessive mitochondrial fission, contributing to mitochondrial dysfunction and apoptosis. Here, we evaluated the inhibitory effect of Bcl‐xl, an antiapoptotic protein, on the action of Mff by using live‐cell fluorescence imaging. Microscopic imaging analysis showed that overexpression of Mff induced mitochondrial fragmentation and apoptosis, which were reversed by coexpression of Bcl‐xl. Microscopic imaging and live‐cell fluorescence resonance energy transfer analysis demonstrated that Bcl‐xl reconstructs the Mff network from punctate distribution of higher‐order oligomers to filamentous distribution of lower‐order oligomers. Live‐cell fluorescence resonance energy transfer two‐hybrid assay showed that Bcl‐xl interacted with Mff to form heterogenous oligomers with 1 : 2 stoichiometry in cytoplasm and 1 : 1 stoichiometry on mitochondria, indicating that two Bcl‐xl molecules primarily interact with four Mff molecules in cytoplasm, but with two Mff molecules on mitochondria.

## Abstract 2

Mitochondrial fission factor (Mff)‐mediated mitochondrial fission is positively correlated with the self‐oligomerization of Mff. Bcl‐xl directly interacts with Mff to prevent Mff‐mediated mitochondrial fission and apoptosis. Bcl‐xl interacts with Mff to form heterogenous hexamers with 1 : 2 stoichiometry in cytoplasm and heterogenous tetramers with 1 : 1 stoichiometry on the mitochondrial membrane, respectively.

## Graphical abstract

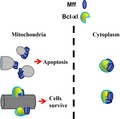

**Abbreviations**
CV: coefficient of variation
CV_CFP_: coefficient of variation value of CFP
Drp1: Dynamin‐related protein 1
*E*_A_: acceptor‐centric FRET efficiency
*E*_D_: donor‐centric FRET efficiency
FRET: fluorescence resonance energy transfer
*I*_AA_: acceptor excitation and acceptor emission
*I*_DA_: donor excited acceptor emission
*I*_DD_: donor excitation and donor emission
mAb: monoclonal antibody
Mff: mitochondrial fission factor
*R*_C_: concentration ratio
ROI: region of interest
SD: standard deviation
YFP‐Bcl‐xl: YFP‐labeled Bcl‐xl

Mitochondria are highly dynamic organelles that continuously divide and fuse in response to cellular signaling and differentiation from ATP production to apoptosis 1, 2, 3. The opposing processes of fission (division) and fusion regulate mitochondrial morphology and function 4. Mitochondrial fission has been linked to the cellular death program of apoptosis 5. The mitochondrial fission factor (Mff), a tail‐anchored membrane protein, induces excessive mitochondrial fission, contributing to mitochondrial dysfunction and apoptosis 6, whereas silencing Mff blocks fission, thus resulting in fused and elongated mitochondria 7, 8. Bcl‐xl, the core antiapoptotic member of the Bcl‐2 family of proteins, increases mitochondrial fission and fusion. However, the regulatory relationship between Bcl‐xl and Mff remains unknown.

Bcl‐xl mainly localizes on mitochondria and inhibits mitochondria‐mediated apoptosis 9, 10. Bcl‐xl was previously implicated in promoting mitochondrial fusion through an interaction with mitofusin protein Mfn2 11 and in inhibiting both mitochondrial fragmentation and cell death mediated by Dynamin‐related protein 1 (Drp1)/Dnm1 12. Cortical neurons that lack Bcl‐xl display mitochondrial fragmentation, and restoring Bcl‐xl leads to elongated mitochondria 13. However, the role of Bcl‐xl in mitochondrial fission was also supported by the interaction of Bcl‐xl with Drp1 in synaptogenesis 14. Mitochondrial recruitment of Drp1 is mainly dependent on Mff 1.

This report uses fluorescence imaging to explore whether Bcl‐xl prevents Mff‐mediated mitochondrial fission and apoptosis in HeLa cells. Microscopic fluorescence imaging of cells expressing CFP‐Mff showed that Mff facilitates mitochondrial fragmentation and apoptosis, and coexpressing Bcl‐xl not only redistributes Mff from punctate distribution to filamentous distribution but also significantly prevents Mff‐mediated mitochondrial fragmentation and apoptosis. Fluorescence resonance energy transfer (FRET) two‐hybrid assay in living cells coexpressing CFP‐Mff and YFP‐Bcl‐xl demonstrates that Bcl‐xl binds to Mff to form heterogenous oligomers with 1 : 2 stoichiometry in cytoplasm and with 1 : 1 stoichiometry on mitochondrial membrane.

## Materials and methods

### Plasmids

YFP‐Bcl‐xl was kindly provided by the Streuli lab 15. GFP‐Mff (#49153), pEYFP‐C1‐Drp1 (#45160), ECFP‐Bak (#31501), pcDNA3‐CFP (CFP; #13030) and pcDNA3‐YFP (YFP; #13033) plasmids were purchased from Addgene Company (Cambridge, MA, USA). The pECFP‐C1 vector and the pEYFP‐C1 vector were respectively obtained by double‐enzyme digestion (XhoI‐BamHI) of ECFP‐Bak plasmids and pEYFP‐C1‐Drp1 plasmids. Mff was obtained by double‐enzyme digestion (XhoI‐BamHI) of GFP‐Mff and then respectively ligated into the XhoI‐BamHI of the pECFP‐C1 vector and the pEYFP‐C1 vector to yield CFP‐Mff and YFP‐Mff plasmids.

### Cell culture and transfection

HeLa cells obtained from the Department of Medicine, Jinan University (Guangzhou, China) were cultured just as described previously 16. When cells reached 70–90% confluence in a 35‐mm glass dish, cells were transfected with indicated amounts of plasmids in the presence or absence of 50 µm Z‐VAD‐FMK (MedChem Express) using TurboFect Transfection Reagent (Thermo Scientific, Waltham, MA, USA) for 24 h.

### Cell staining

The transfected cells were light washed one time with PBS and then incubated with culture medium of Hoechst 33258 (Sigma‐Aldrich, St. Louis, MO, USA) and DilC1(5) (Invitrogen, Carlsbad, CA, USA), or MitoTracker Deep Red 633 dye (Molecular Probes) according to the manufacturer’s protocol in the presence of 50 µm Z‐VAD‐FMK for 30 min at room temperature.

### Antibodies

The following primary antibodies were used in this study: rabbit monoclonal antibody (mAb) anti‐Bcl‐xl serum 54H6 (#2764; Cell Signaling Technology, Danvers, MA, USA), polyclonal rabbit anti‐Mff serum (17090‐1‐AP; Proteintech, Inc., Rosement, IL, USA), mouse mAb anti‐β‐tubulin serum (HC101; TransGen Biotech, Beijing, China) and glyceraldehyde‐3 phosphate dehydrogenase Mouse mAb(2B8) (TDY042; TDY Biotech, Beijing, China). Secondary antibodies used for western blotting include horseradish peroxidase AffiniPure goat anti‐rabbit IgG (H + L; S004; TDY Biotech, Beijing, China) and horseradish peroxidase AffiniPure goat anti‐mouse IgG (H + L; S001; TDY Biotech, Beijing, China). Antibodies were used according to the manufacturer’s recommendations. Tubulin and glyceraldehyde‐3 phosphate dehydrogenase were used as loading control.

### Microscopy

Images were acquired using a zen 2.3 software version Carl Zeiss Microscope (Carl Zeiss, Oberkochen, Germany) equipped with or without ApoTome.2 modules and a 63× 1.4 NA oil immersion lens, as well as a charge‐coupled device camera (Axiocam 506 mono; Carl Zeiss). A cube comprising a BP436/20 excitation filter (Carl Zeiss) and a dichroic mirror of DFT 455 (Carl Zeiss), as well as a BP480/40 emission filter (Carl Zeiss), was used for donor excitation and donor emission (*I*_DD_) imaging; a cube comprising a BP500/20 excitation filter (Carl Zeiss) and a dichroic mirror of DFT 515 (Carl Zeiss), as well as a BP535/30 emission filter (Carl Zeiss), was used for acceptor excitation and acceptor emission (*I*_AA_) imaging; and a cube comprising a BP436/20 excitation filter (Carl Zeiss) and a dichroic mirror of DFT 455 (Carl Zeiss), as well as a BP535/30 emission filter (Carl Zeiss), was used for FRET imaging [donor excited acceptor emission (*I*_DA_)].

### FRET two‐hybrid assay

FRET microscopy is a powerful tool for exploring the association and disassociation of intracellular macromolecular complexes during signal transduction in living cells 17, 18. Donor‐centric FRET efficiency (*E*_D_) and acceptor‐centric FRET efficiency (*E*_A_), as well as the concentration ratio (*R*_C_) of acceptor to donor, were measured as follows 19, 20:

(1) $$E_{D}=\frac{F_{C}}{F_{C}+{\text{GI}}_{\text{DD}}}$$

(2) $$E_{A}=\frac{F_{C}}{aI_{\text{AA}}}\frac{\varepsilon_{A}\left(\text{FRET}\right)}{\varepsilon_{D}\left(\text{FRET}\right)}$$

(3) $$R_{C}=\frac{kI_{\text{AA}}}{F_{C}/G+I_{\text{DD}}}$$

where

(4) $$F_{C}=I_{\text{DA}}-a\left(I_{\text{AA}}-cI_{\text{DD}}\right)-d\left(I_{\text{DD}}-bI_{\text{AA}}\right)$$

where *ε*_A_ and *ε*_D _are the absorption coefficient of acceptor (A) and donor (D), respectively; *F*_C_ is sensitized emission fluorescence; *a* and *b* are acceptor bleed‐through in the *I*_DA_ and *I*_DD_ filter sets, and *c* and *d* are donor bleed‐through in the *I*_AA_ and *I*_DA_ filter sets, respectively; *G* is the ratio of the sensitized emission of acceptor to an equivalent quenching of donor; and *k* is the ratio of donor/acceptor fluorescence intensity for equimolar concentrations in the absence of FRET.

The stoichiometry (*v*) of a D–A complex is 21

(5) $$v=\frac{n_{A}}{n_{D}}=\frac{E_{D,max}}{E_{\text{A,max}}}$$

where *E*_A,max_ (slope) was determined by linearly fitting the *E*_D_–*R*_C_ plot, and *E*_D,max_ (slope) was determined by linearly fitting the *E*_A_–1/*R*_C_ plot 22:

(6) $$E_{D}={\mathrm{EC}}_{A,max}$$

(7) $$E_{A}=E_{\text{D,max}}\frac{1}{R_{C}}$$

### Statistics

Data were presented as mean ± standard deviation (SD) from three independent experiments. The Student’s *t*‐test was used to evaluate the significance of difference between two groups. A *P*‐value *<*0.05 was defined as a statistically significant difference. Image analysis was conducted with zen 2.3 software (Carl Zeiss). Statistical and graphic analyses were done using the software spss 19.0 (SPSS, Chicago, IL, USA) and Origin 8.0 (OriginLab Corporation, Northampton, MA, USA).

## Results

### Mff facilitates mitochondrial fragmentation

**Figure 1:**
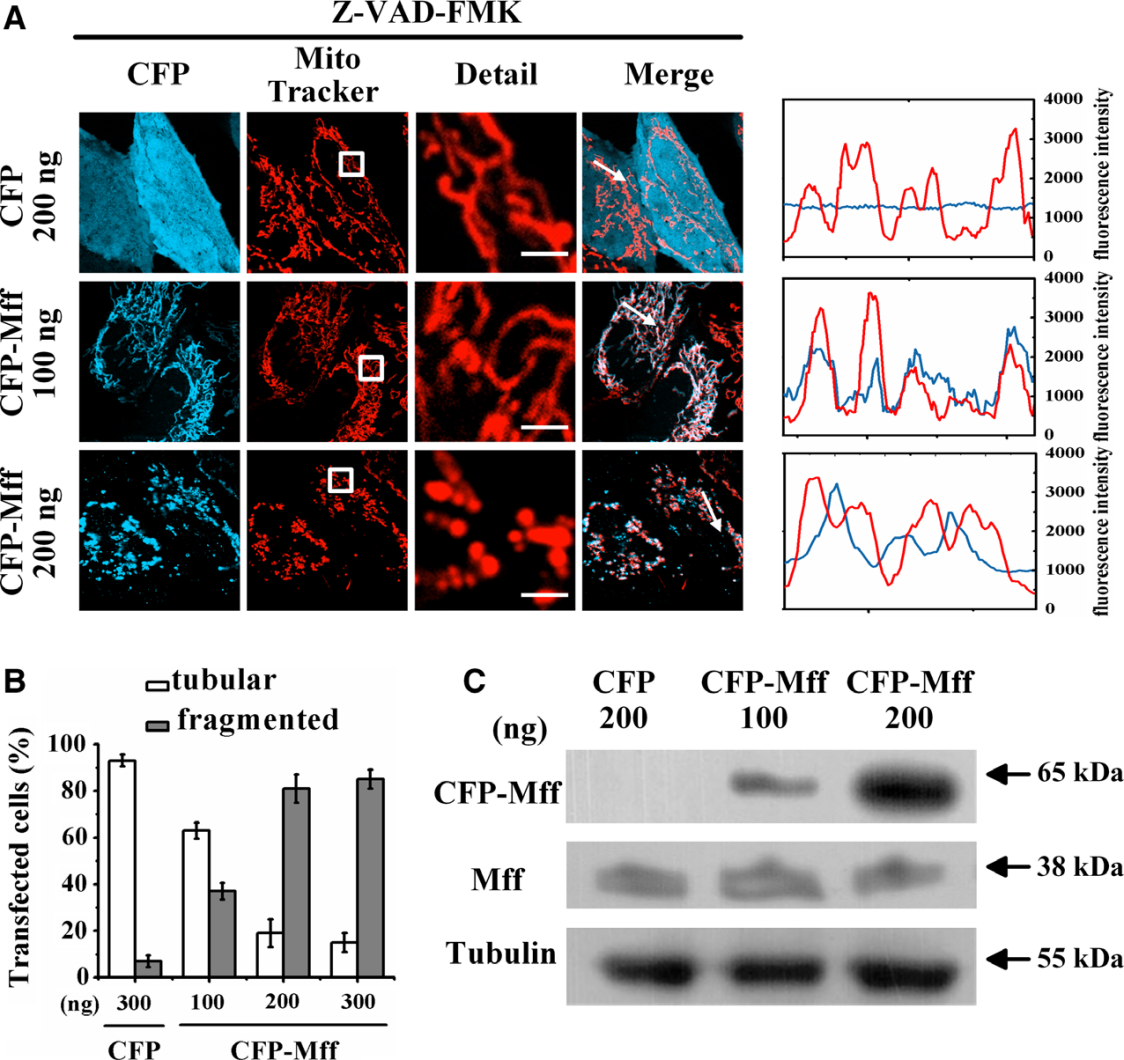
Mff facilitates mitochondrial fragmentation. (A) Fluorescence microscopic images of living cells transfected with the indicated amounts of CFP (empty vector) or CFP‐Mff and also stained with MitoTracker. All treatments were carried out in the presence of 50 μm Z‐VAD‐FMK for inhibiting apoptosis. Scale bars: 2 μm. Line scans show the fluorescence intensities of CFP signals (cyan) and mitochondria stained by MitoTracker (red) along the selected white arrows in the merge panels. (B) Percentages of cells with indicated mitochondrial morphologies in CFP‐positive cells (*n* = 150) transfected with the indicated amounts of plasmids. Data were collected from three independent experiments. The error bars represent SD. (C) Expression level of Mff in HeLa cells transfected with the indicated amounts of CFP (empty vector) or CFP‐Mff assessed by western blotting.

To examine the effect of Mff on mitochondrial morphology, we transfected cells with CFP (control) or CFP‐labeled Mff (CFP‐Mff). All cells were treated with Z‐VAD‐FMK, a pan‐caspase inhibitor, for inhibiting apoptosis and also stained with MitoTracker for labeling mitochondria. Fluorescence microscopic images showed that mitochondria in cells expressing CFP were tubular (Fig. 1A, upper panel), whereas most mitochondria in cells transfected with 200 ng of CFP‐Mff were fragmented, and Mff mainly exhibited punctate distribution and was located at the end of fragmented mitochondria (Fig. 1A, lower panel). Statistical results showed that approximately 40% of cells transfected with 100 ng of CFP‐Mff exhibited mitochondrial fragmentation, whereas cells transfected with 200 or 300 ng of CFP‐Mff showed about 80% of cells with mitochondrial fragmentation (Fig. 1B). Furthermore, to further clarify the effect of Bcl‐xl on mitochondrial morphology, we used western blotting to detect the expression level of Mff in cells transfected with the indicated amounts of plasmids, and found that the expression level of Mff in cells transfected 200 ng of CFP‐Mff was significantly higher than that of the control group (Fig. 1C). These experiments demonstrated that overexpression of Mff facilitates mitochondrial fragmentation. Transfection of 200 ng of CFP‐Mff was adopted in the following experiments without specific indication.

### Mff self‐oligomerizes in cytoplasm and mitochondria

**Figure 2:**
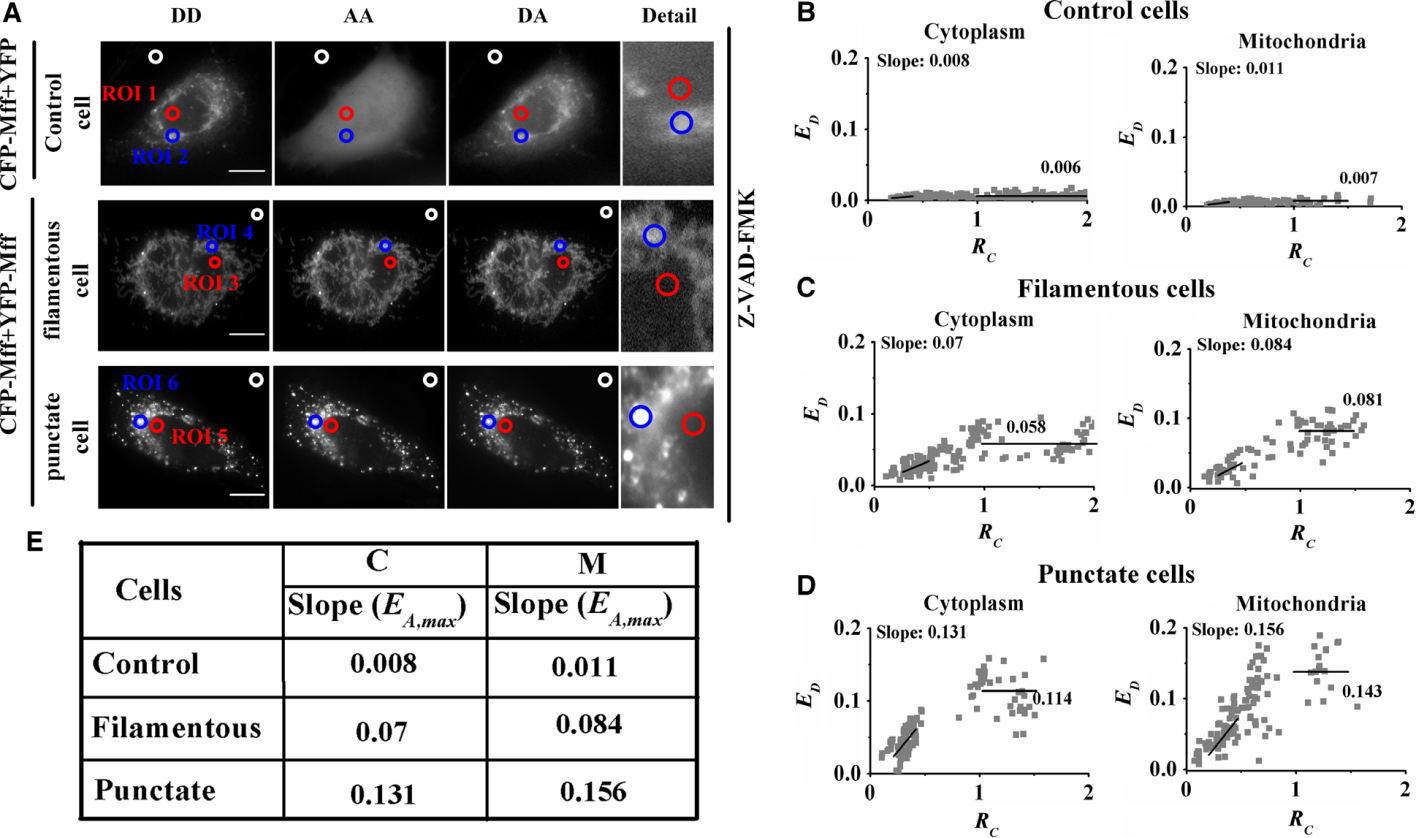
Mff oligomerizes in cytoplasm and mitochondria. (A) Three fluorescence images (DD channel, DA channel, and AA channel; fluorescence microscope without the ApoTome.2 module) of living cells separately coexpressing CFP‐Mff + YFP (upper panel) or CFP‐Mff + YFP‐Mff (lower panel). All treatments were carried out in the presence of 50 μm Z‐VAD‐FMK. Scale bars: 10 μm. (B) *E*_D_–*R*_C_ plots in cytoplasm (left) and mitochondrial regions (right) from about 60 control living cells. Each gray dot symbol corresponds to data from an ROI. (C, D) Similar to (B) from living cells with filamentous CFP‐Mff and YFP‐Mff, or punctate CFP‐Mff and YFP‐Mff distribution. (E) Summarizing data of *E*_A,max_ values from the slope of *E*_D_–*R*_C_ plots in (B)–(D).

To test whether Mff forms homo‐oligomers, we cotransfected cells with 100 ng of CFP‐Mff (donor) and 100 ng of YFP‐Mff (acceptor). All cells were treated with Z‐VAD‐FMK. The cells were imaged using three different fluorescence filter cubes for quantitative FRET two‐hybrid assay according to Eqns ((1), (2), (3)): the donor channel (*I*_DD_), the acceptor channel (*I*_AA_) and the FRET channel (*I*_DA_). In cells coexpressing CFP‐Mff and YFP (control), we circumscribed the nuclear area as cytoplasm [region of interest 1 (ROI 1)], punctate Mff area as mitochondrial region (ROI 2) and noncells area as background region (the white circle), respectively (Fig. 2A, upper panel). The measured *E*_D_ and *R*_C_ were 0.007 and 1.003 for ROI 1, and 0.008 and 0.711 for ROI 2. We repeated FRET two‐hybrid assay method from at least 60 cells with different *R*_C _and obtained the corresponding *E*_D_–*R*_C_ plot (Fig. 2B). Linearly fitting the *E_D_*–*R*_C_ plot (*R*_C_ < 0.4) obtained a slope of 0.008 (*E*_A,max_) according to Eqn (6) and also obtained an *E*_D,max_ value of 0.006 by averaging the *E*_D_ values in the 1–2 range of *R*_C_ for cytoplasm (Fig. 2B, left). Linearly fitting the *E*_D_–*R*_C_ plot (*R*_C_ < 0.4) obtained a slope of 0.011 (*E*_A,max_) and also obtained an *E*_D,max_ value of 0.007 by averaging the *E*_D_ values in the 1–1.5 range of *R*_C _for mitochondrial regions (Fig. 2B, right), indicating no interaction between CFP‐Mff and YFP.

Similarly, FRET two‐hybrid assay was performed for cells with filamentous CFP‐Mff and YFP‐Mff (Fig. 2A, middle panel, and Fig. 2C) or with punctate CFP‐Mff and YFP‐Mff (Fig. 2A, lower panel, and Fig. 2D) distribution. The measured *E*_A,max_ values between CFP‐Mff and YFP‐Mff in cytoplasm (C) and mitochondrial regions (M) were larger than 0.07 (Fig. 2E), which was much higher than the 0.011 of control cells, indicating the oligomerization of intracellular Mff.

### Bcl‐xl facilitates mitochondrial fusion

**Figure 3:**
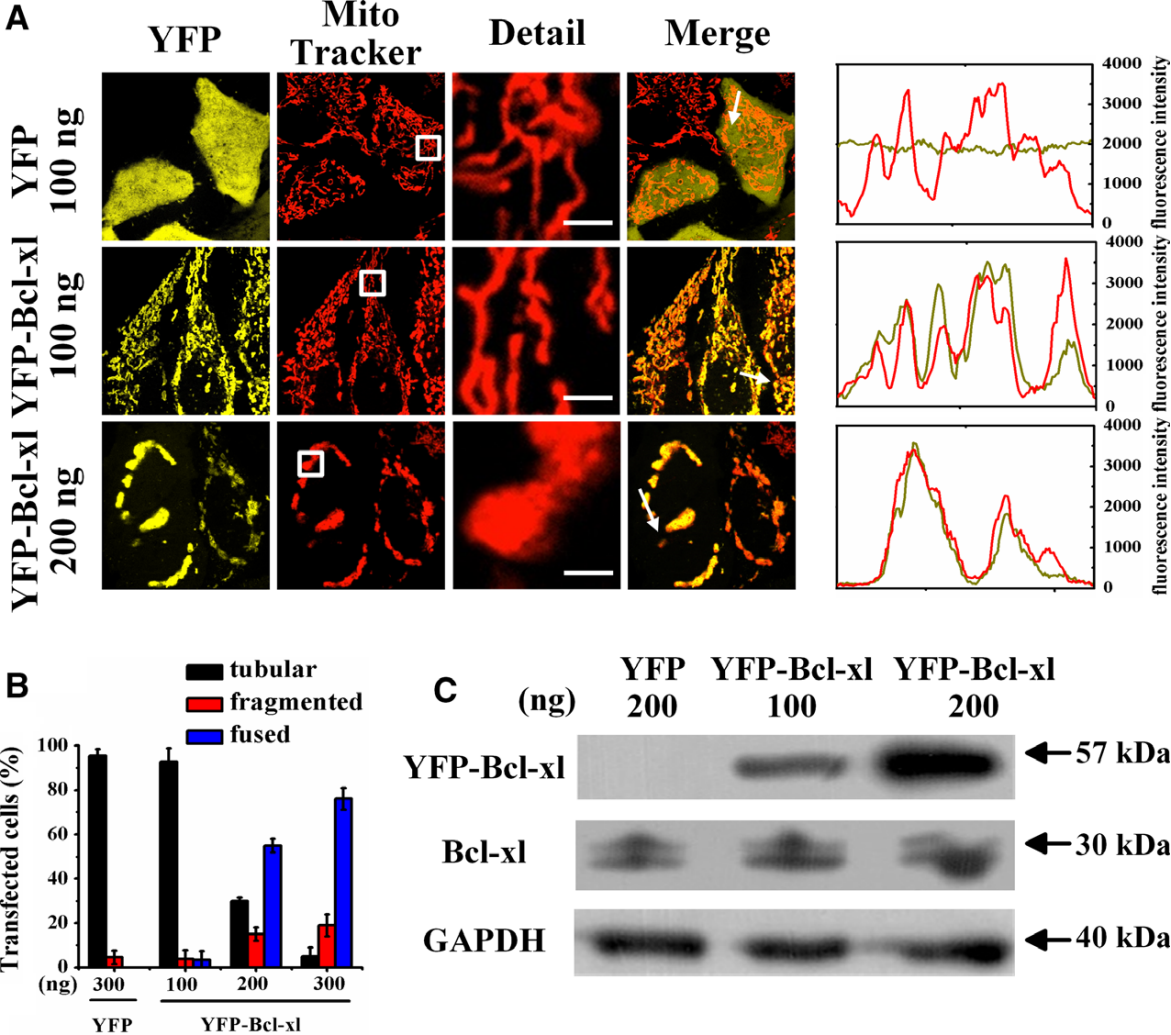
Bcl‐xl reconstructs the mitochondrial network. (A) Fluorescence microscopic images of living cells transfected with the indicated amounts of YFP (empty vector) or YFP‐Bcl‐xl plasmids, and also stained with MitoTracker. Scale bars: 2 μm. Line scans show the fluorescence intensities of YFP signals (yellow) and mitochondria stained by MitoTracker (red) along the selected white arrows in the merge panels. (B) Percentages of cells with indicated mitochondrial morphologies in YFP‐positive cells (*n* = 150) transfected with the indicated amounts of plasmids. Data were collected from three independent experiments. The error bars represent SD. (C) Expression level of Bcl‐xl in cells transfected with the indicated amounts of YFP (empty vector) or YFP‐Bcl‐xl assessed by western blotting.

To explore the effect of Bcl‐xl on mitochondrial morphology, we transfected cells with YFP (control) or YFP‐labeled Bcl‐xl (YFP‐Bcl‐xl) and also stained them with MitoTracker. Fluorescence microscopic images showed that mitochondria in the cells transfected with 100 ng of YFP‐Bcl‐xl were tubular, consistent with mitochondria in the cells expressing YFP (Fig. 3A, upper panel), and Bcl‐xl exhibited good colocalization with the tubular mitochondrial membrane (Fig. 3A, middle panel). However, cells transfected with 200 ng of YFP‐Bcl‐xl showed that most mitochondria were fused, and Bcl‐xl mainly exhibited clustered distribution and good colocalization with fused mitochondria (Fig. 3A, lower panel). Statistical results showed that about 90% of cells transfected with 100 ng of YFP‐Bcl‐xl exhibited tubular mitochondria, whereas more than 50% of cells transfected with 200 ng of YFP‐Bcl‐xl exhibited fused mitochondria (Fig. 3B). Furthermore, we also performed western blotting assay to assess the expression level of Bcl‐xl in HeLa cells transfected with the indicated plasmids (Fig. 3C). The experimental results showed that the expression level of Bcl‐xl in cells transfected with 200 ng of YFP‐Bcl‐xl was significantly higher than that of the control group. These experiments demonstrate that overexpression of Bcl‐xl mainly facilitated mitochondrial fusion. Transfection of 80 ng of Bcl‐xl was adopted in the following experiments without specific indication.

### Bcl‐xl prevents Mff‐mediated mitochondrial fission and apoptosis

**Figure 4:**
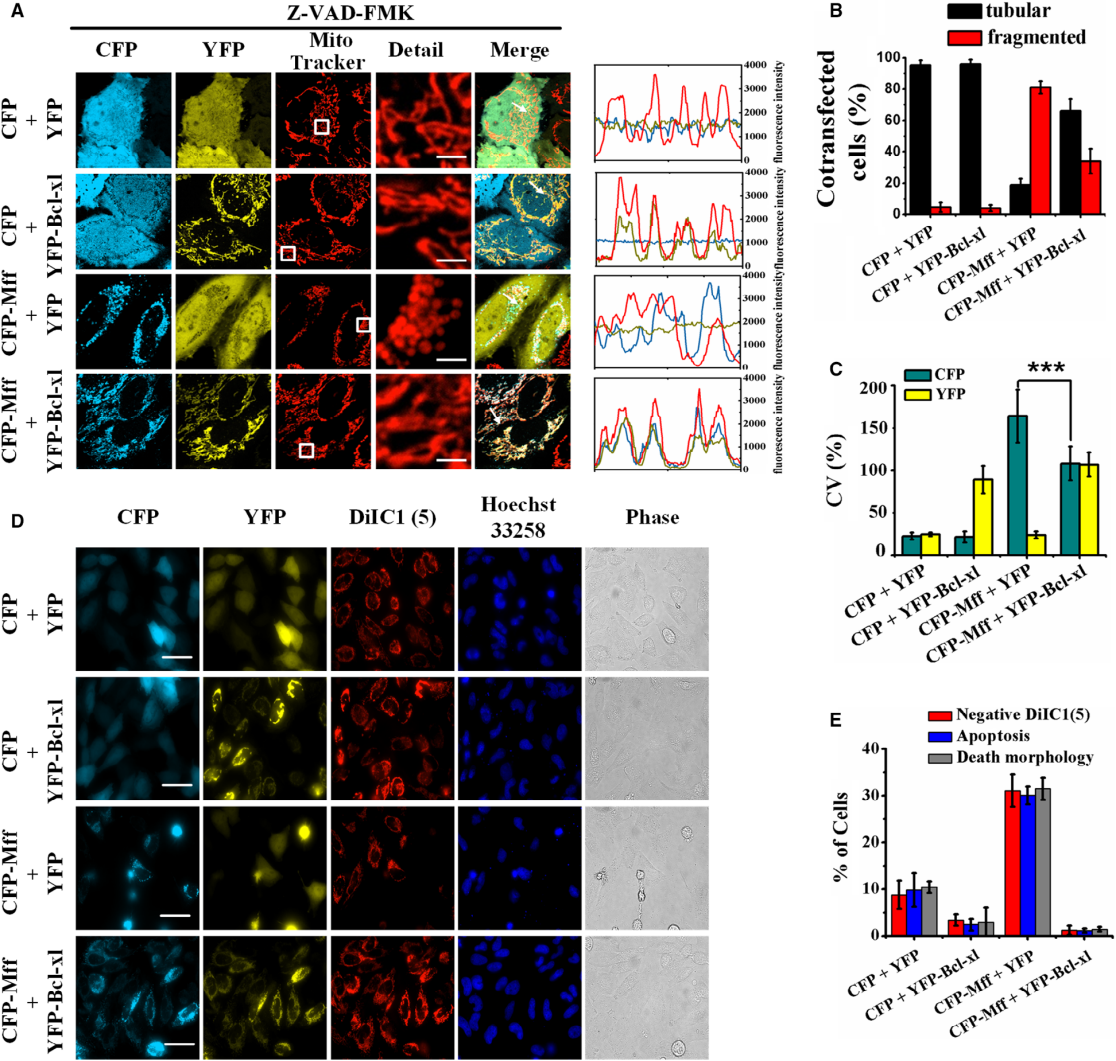
Inhibition of Mff‐mediated mitochondrial fission and apoptosis by Bcl‐xl. (A) Fluorescence microscopic images of living cells coexpressing CFP and YFP, CFP and YFP‐Bcl‐xl, CFP‐Mff and YFP, and CFP‐Mff and YFP‐Bcl‐xl, respectively, and also stained with MitoTracker. All treatments were carried out in the presence of 50 μm Z‐VAD‐FMK. Scale bars: 2 μm. Line scans show the fluorescence intensities of CFP signals (cyan) and YFP signals (yellow), as well as mitochondria stained by MitoTracker staining (red), along the selected white arrows in the merge panels. (B) Percentages of cells with indicated mitochondrial morphologies collected from 150 cells in each of three independent experiments. The error bars represent SD. (C) CV of CFP and YFP obtained from 100 cells in each of three independent experiments. CV_CFP_ = SD_CFP_/mean_CFP_ and CV_YFP_ = SD_YFP_/mean_YFP_. The error bars represent SD_CV_. The Student’s *t*‐test was used to evaluate the significance of difference between two groups. ***P < 0.001. (D) Fluorescence microscopic images (fluorescence microscope without the ApoTome.2 module) of living cells coexpressing CFP and YFP, CFP and YFP‐Bcl‐xl, CFP‐Mff and YFP, and CFP‐Mff and YFP‐Bcl‐xl, respectively, and double stained with DilC1(5) and Hoechst 33258. Scale bars: 50 μm. (E) Percentages of cells (*n* = 150) with mitochondrial membrane potential loss [negative DiIC1(5)] and karyopyknosis, as well as edge shrinkage (death morphology). Data were collected from three independent experiments. The error bars represent SD.

Given that Bcl‐xl maintains mitochondrial morphology (Fig. 3A, middle panel), we wondered whether Bcl‐xl prevents Mff‐mediated mitochondrial fission and apoptosis. To answer this question, we evaluated mitochondrial morphology and the distribution of Mff in living cells coexpressing CFP‐Mff and YFP in the presence or absence of Bcl‐xl. Fluorescence microscopic images showed that Mff was mainly filamentous and had a good colocation with Bcl‐xl in the cells with tubular mitochondria (Fig. 4A, fourth row). Statistical results showed that about 80% of the cells coexpressing CFP‐Mff and YFP, but only 30% of the cells coexpressing CFP‐Mff and YFP‐Bcl‐xl exhibited fragmented mitochondria (Fig. 4B), demonstrating that Bcl‐xl prevented Mff‐induced mitochondrial fragmentation. Mff located mainly in punctate at the mitochondrial endpoint in the cells coexpressing CFP‐Mff and YFP, whereas Mff mainly colocalized with the tubular mitochondria in the cells coexpressing CFP‐Mff and YFP‐Bcl‐xl (Fig. 4A), indicating that Bcl‐xl affected the location of Mff from punctate distribution to filamentous distribution, which was further verified by the higher coefficient of variation value of CFP (CV_CFP_) (1.64) of the cells coexpressing CFP‐Mff and YFP than the 1.01 of the cells coexpressing CFP‐Mff and YFP‐Bcl‐xl (Fig. 4C). Interestingly, the almost identical CV values of both CFP and YFP in the cells coexpressing CFP‐Mff and YFP‐Bcl‐xl (Fig. 4C) further demonstrate the redistribution of Mff by Bcl‐xl.

It is reported that Mff not only promotes mitochondrial fission but also mediates apoptosis 23, 24. To explore whether Bcl‐xl inhibits Mff‐mediated apoptosis, we cotransfected cells with CFP and YFP, CFP and YFP‐Bcl‐xl, CFP‐Mff and YFP, and CFP‐Mff and YFP‐Bcl‐xl, respectively. All cells were double stained with DiIC1(5) and Hoechst 33258. Mitochondrial membrane potential, nuclear shape and cellular morphology were imaged synchronously by fluorescence microscopy. Cells coexpressing CFP and YFP exhibited no obvious signs of apoptosis (Fig. 4D, first row), and cells coexpressing CFP‐Mff and YFP exhibited typical apoptosis characteristics: mitochondrial membrane potential loss from the image of DiIC1(5) channel, karyopyknosis from the image of Hoechst 33258 channel and edge shrinkage from the brightfield image (Fig. 4D, third row), which were obviously inhibited by coexpression of Bcl‐xl (Fig. 4E), suggesting that Bcl‐xl prevented Mff‐mediated apoptosis.

### Bcl‐xl binds to Mff

**Figure 5:**
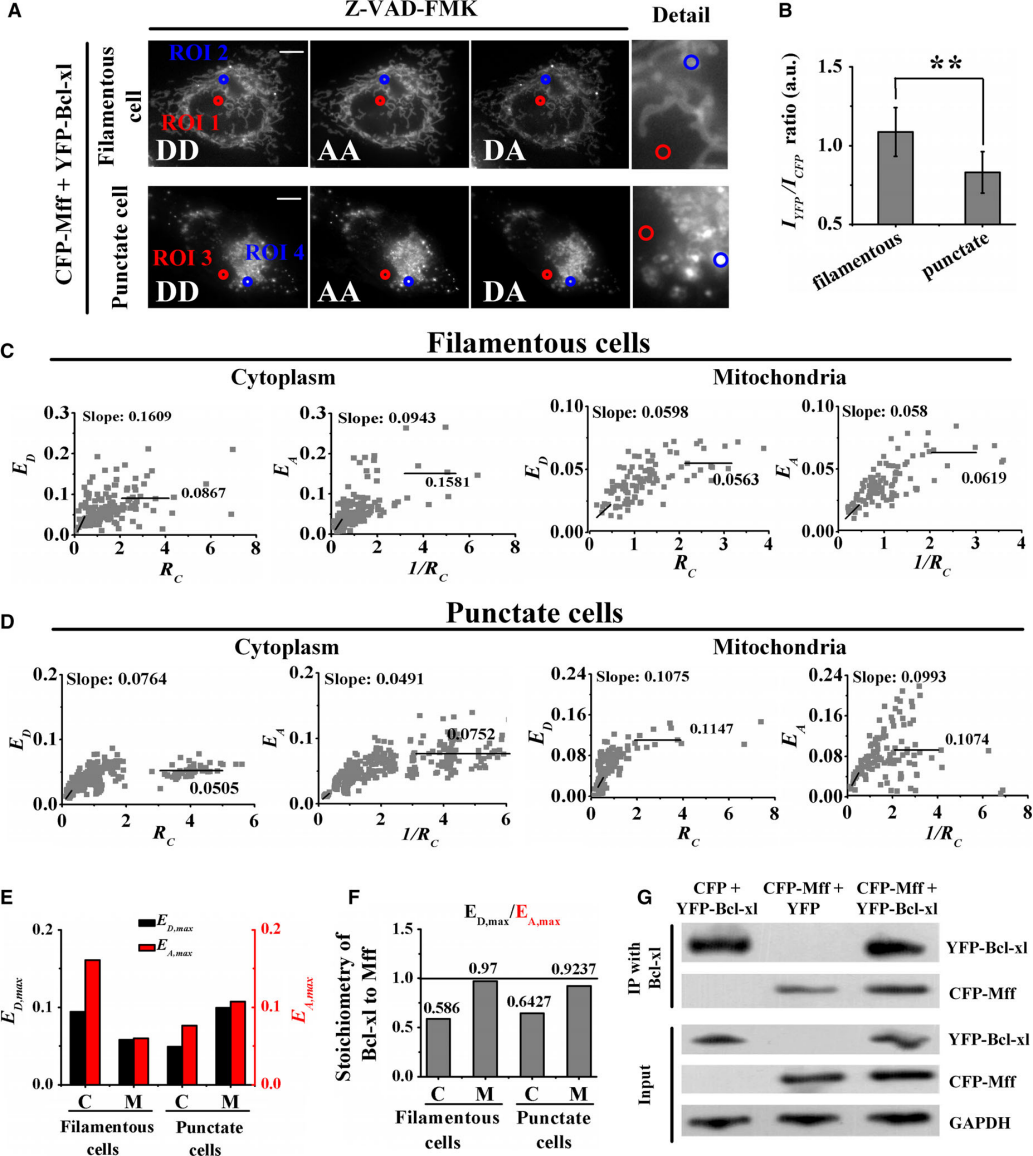
Interaction between Bcl‐xl and Mff. (A) Fluorescence images of living cells coexpressing CFP‐Mff and YFP‐Bcl‐xl in DD, DA and AA channels (fluorescence microscope without the ApoTome.2 module), respectively. All treatments were carried out in the presence of 50 μm Z‐VAD‐FMK. Scale bars: 20 μm. (B) The statistical results of the *I_YFP_*/*I_CFP_* ratio from at least 100 cells with filamentous Mff and Bcl‐xl or punctate Mff and Bcl‐xl distribution, respectively. Data were collected from three independent experiments. The error bars represent SD. The Student’s *t*‐test was used to evaluate the significance of difference between two groups. **P < 0.01. (C) The *E*_D_–*R*_C_ plot (first and third panels) and *E*_A_–*1/R*_C_ plot (second and fourth panels) of the cytoplasm and tubular mitochondrial area from 70 cells coexpressing Mff and Bcl‐xl. (D) Similar to (C) for living cells with punctate CFP‐Mff and YFP‐Mff distribution. (E) Bars depict the slope from the corresponding *E*_A_–*1/R*_C_ plot (*E*_D,max_; black) and the slope from the corresponding *E*_D_–*R*_C_ plot (*E*_A,max_; red) for cytoplasm (C) and mitochondrial area (M). (F) Stoichiometric ratio (*v = E*_D,max_ */E*_A,max_) of Bcl‐xl/Mff complexes. (G) Coimmunoprecipitation to further verify the direct interaction between Mff and Bcl‐xl. After transfection with the indicated expression constructs, cell lysates were subjected to immunoprecipitation with anti‐Bcl‐xl serum, and the precipitated complexes were analyzed by western blot with indicated antibodies.

Based on the finding that Mff colocates with Bcl‐xl on mitochondria (Fig. 4A, fourth row), we wondered whether Bcl‐xl forms a complex directly with Mff. For this purpose, we performed quantitative FRET measurements in living cells coexpressing CFP‐Mff and YFP‐Bcl‐xl. Figure 5A shows the fluorescence images of cells with filamentous Mff and Bcl‐xl distribution (upper panel) or punctate Mff and Bcl‐xl distribution (lower panel) in donor (DD), acceptor (AA) and FRET (DA) channels. To explore whether the tubular or fragmented mitochondrial distribution associates with the relative expression level between Bcl‐xl and Mff, we measured the emission intensity ratio (*I*_YFP_/*I*_CFP_) of [acceptor]/[donor] of living cells to evaluate the relative expression level of Bcl‐xl and Mff. The statistical *I*_YFP_/*I*_CFP_ ratio of the cells with filamentous Mff and Bcl‐xl distribution was 1.087, which was much higher than the 0.83 of the cells with punctate Mff and Bcl‐xl distribution (Fig. 5B), indicating that the inhibitory action of Bcl‐xl on Mff‐mediated mitochondrial fission was positively correlated with the relative expression level between Bcl‐xl and Mff.

As shown in Fig. 5A, in the cells with filamentous Mff and Bcl‐xl distribution, we chose the nuclear area as cytoplasm (ROI 1) and the filamentous area as the tubular mitochondrial region (ROI 2). In the cells with punctate Mff and Bcl‐xl distribution, we also chose the nuclear area as cytoplasm (ROI 3) and the punctate area as the fragmented mitochondrial region (ROI 4). Next, we performed quantitative E‐FRET and 3^3^‐FRET measurements for the indicated ROIs in the two kinds of cells to obtain *E*_D_ and *E*_A_ values, as well as the corresponding *R*_C_. The measured *E*_D_ and *E*_A _values were 0.086 and 0.151 for the ROI 1, 0.067 and 0.071 for the ROI 2, 0.041 and 0.072 for the ROI 3, and 0.109 and 0.115 for the ROI 4. We reperformed quantitative E‐FRET and 3^3^‐FRET measurements in cells with different *R*_C_ values, and the corresponding *E*_D_–*R*_C_ (first and third panels) and *E*_A_–*1/R*_C _(second and fourth panels) plots are shown in Fig. 5C,D. According to Eqns (6,7), linearly fitting the *E*_D_–*R*_C _plots (*R*_C_: <0.5) and *E*_A_–1/*R*_C_ plots (1/*R*_C_: 0.25–0.5) (Fig. 5C,D) obtained the *E*_D,max_ and *E*_A,max_ values in cytoplasm (C) and mitochondrial (M) regions of cells (Fig. 5E). According to Eqn (5), the stoichiometry (*E*_A,max_/*E*_D,max_) of the Bcl‐xl/Mff complex was 0.586 (0.0943/0.1609) in the cytoplasm of filamentous cells, 0.97 (0.058/0.0598) in the tubular mitochondrial membrane of filamentous cells, 0.6427 (0.0491/0.0764) in the cytoplasm of punctate cells and 0.9237 (0.0993/0.1075) in the fragmented mitochondrial membrane of punctate cells (Fig. 5F). It is of particular relevance to note that the mitochondrial regions we circled probably included the cytoplasm. Therefore, the actual stoichiometry of the Bcl‐xl/Mff complex in mitochondria may be slightly higher than what we measured.

To further confirm the direct interaction between Bcl‐xl and Mff, coimmunoprecipitation for exogenous proteins was performed in cells cotransfected with the indicated plasmids. As shown in Fig. 5G, the interaction between Bcl‐xl and Mff was observed in the cells cotransfected with CFP‐Mff and YFP, or CFP‐Mff and YFP‐Bcl‐xl. The interaction was completely abolished in the cells cotransfected with CFP and YFP‐Bcl‐xl.

## Discussion

Our results further confirm the notion that Mff facilitates mitochondrial fragmentation. Under transfection with 200 or 300 ng of Mff, the percentage of the cells with fragmented mitochondria was about 80% (Fig. 1B), consistent with the previous finding that more than 80% of cells had extensively fragmented mitochondria irrespective of the Mff expression level 1. However, only 40% of cells expressing Mff exhibited obvious fragmented mitochondria under 100 ng of CFP‐Mff transfection (Fig. 1B), indicating that Mff induced expression level–dependent mitochondrial fragmentation. In addition, Mff precisely colocalized with mitochondria in the cells with tubular mitochondria (Fig. 1A, middle panel), consistent with the finding by Gandre‐Babbe and van der Bliek 23. We also found that Mff was positioned at the mitochondrial endpoint in the cells with fragmented mitochondria (Fig. 1A, lower panel), which may be the constriction and scission sites of the mitochondrial membrane 1, 25

Localization of Mff in both mitochondria and cytoplasm (Fig. 1A), consistent with *in vitro* findings by using western blots analysis 23, indicate that Mff may shuttle between the mitochondrial membrane and cytoplasm to maintain a dynamic balance or transport other proteins. In the cells expressing the Mff mutant lacking the transmembrane domain, Mff was dispersed in the cytoplasm, and fragmented mitochondria were rarely detected 1, indicating that Mff localization on mitochondrial is a prerequisite for Mff‐induced mitochondrial fragmentation. Based on these experimental results, it is not difficult to speculate that accumulation and oligomerization of Mff on mitochondria is required for mitochondrial fragmentation.

Our live‐cell FRET analysis shows that Mff forms homo‐oligomers in the cytoplasm and mitochondria (Fig. 2), which supports the *in vitro* findings by using western blots analysis 23. Cells with fragmented mitochondria had a higher *E*_A,max_ or *E*_D,max_ value between CFP‐Mff and YFP‐Mff than that of cells with tubular mitochondria (Fig. 2E), which may be because of the conformation alternation of Mff complexes or formation of higher‐order Mff oligomers. Furthermore, the higher *E*_max_ value on the mitochondrial membrane than that in the cytoplasm (Fig. 2E) indicates the higher oligomeric degree of Mff on mitochondria.

Our data that Bcl‐xl overexpression facilitates mitochondrial fusion to form fused mitochondria (Fig. 3) are consistent with previous findings 11. Transfection with 100 ng of Bcl‐xl had little effect on mitochondrial morphology, whereas 300 ng of YFP‐Bcl‐xl transfection markedly induced mitochondrial fusion (Fig. 3B), further verifying the dose‐dependent effect of Bcl‐xl on mitochondrial networks 11.

Our observation that expression of CFP‐Mff induced about 30% of apoptosis (Fig. 4E) further proves the proapoptotic ability of Mff. Previous studies have suggested that Mff small interfering RNA strongly inhibits cytochrome *c* release from mitochondria in the majority of cells treated with staurosporine 23. Furthermore, Zhou *et al*. 8 reported that excessive mitochondrial fission via Mff upregulation contributed to the disruption of mitochondrial structure and function, as well as mitochondrial apoptosis in cardiomyocytes, which was reversed by loss of Mff.

How does Bcl‐xl prevent the proapoptotic function of Mff? Previous studies have suggested that excessive fission is accompanied by cellular damage 26, 27. It was reported that the ability of Mff to promote mitochondrial fission is strictly dependent on Drp1 1, and the coimmunoprecipitation results showed that Drp1 bound Bcl‐xl and Mff to form the Bcl‐xl/Drp1/Mff complex 28. Our FRET analysis showed that the 0.1 *E* value between CFP‐Mff and YFP‐Bcl‐xl was larger than the 0.01 of control (Fig. 5E), suggesting the potential direct interaction between Mff and Bcl‐xl, which was further verified by coimmunoprecipitation assay (Fig. 5G). According to our data that the CV_CFP_ value in the cells coexpressing CFP‐Mff and YFP‐Bcl‐xl was lower than that in the cells coexpressing CFP‐Mff and YFP (Fig. 4D), we inferred that Bcl‐xl prevented the proapoptotic function of Mff by depolymerizing the higher‐order oligomeric Mff or impeding further oligomerization of Mff. It is also possible that Bcl‐xl impedes the recruitment ability of Mff for Drp1 to prevent Mff‐mediated mitochondrial fission.

Approximate 1 : 2 stoichiometry of the Bcl‐xl/Mff complex in cytoplasm (Fig. 5F) may be caused by the binding of two Bcl‐xl molecules with four Mff molecules. Coimmunoprecipitation, gel filtration and crosslinking assay suggest that cytosolic Bcl‐xl exists as a homodimer 29, 30. FRET analysis in living cells coexpressing CFP‐Mff and YFP‐Mff showed that Mff existed in homo‐oligomers (Fig. 2). In addition, size exclusion chromatography with multiangle light scattering assay in solution showed that Mff lacking its transmembrane segment existed as a stable tetramer 31. Therefore, Bcl‐xl homodimers may interact directly with Mff homotetramers to form hexamers with 1 : 2 stoichiometry in cytoplasm.

The 1 : 1 stoichiometric ratio of the Bcl‐xl/Mff complex on mitochondria (Fig. 5F) may be caused by the binding of two Bcl‐xl molecules with two Mff molecules. Although the C‐terminal transmembrane domain and the N terminus of Bcl‐xl were helpful for its mitochondrial outer membrane targeting 29, 32, the C‐terminal tail of Bcl‐xl is not essential for membrane insertion 32, 33, 34. Previous evidence indicates that Bcl‐xl also targets to the mitochondrial inner membrane 9, and the N terminus of Bcl‐xl may be one component of targeting the mitochondrial inner membrane 32. When the N terminus of Bcl‐xl is inserted into the mitochondria, Bcl‐xl may expose its C‐terminal tail in the cytoplasm to bind the N terminus of Mff. According to the 1 : 2 stoichiometry in cytoplasm and the 1 : 1 stoichiometry in mitochondria of the Bcl‐xl/Mff complex (Fig. 5F), we suspect that Bcl‐xl, in cytoplasm, may interact with Mff to form hetero‐oligomers not only through the binding of the C‐terminal tail but also through the N‐terminal adjacent region of Bcl‐xl with the N‐terminal region of Mff, but in mitochondria only through the C‐terminal tail of Bcl‐xl with the N‐terminal region of Mff. Therefore, two Bcl‐xl molecules interact mainly with four Mff molecules in cytoplasm, but with two Mff molecules on the mitochondrial outer membrane.

## Conclusions

Bcl‐xl prevents Mff‐mediated mitochondrial fission and apoptosis. Mff exists mainly as multimer formation in cytoplasm and mitochondria. Mff‐mediated mitochondrial fission is positively correlated with its self‐oligomerization degree. Live‐cell FRET two‐hybrid assay illustrates that Bcl‐xl directly interacts with Mff, and two Bcl‐xl molecules interact with multiple (possibly four) Mff molecules in the cytoplasm, but with two Mff molecules on mitochondria to form Bcl‐xl/Mff complexes.

## Conflict of interest

The authors declare no conflict of interest.

## Author contributions

YM, XW and TC conceived and supervised the study. YM and MD designed experiments. YM performed experiments. MD, ZM and CZ provided new tools and reagents. YM, MD, FY and CZ analyzed data. YM, BW and TC wrote the manuscript. YM, ZM, CZ, WQ and BW made manuscript revisions.
